# The cell morphogenesis *ANGUSTIFOLIA* (*AN*) gene, a plant homolog of CtBP/BARS, is involved in abiotic and biotic stress response in higher plants

**DOI:** 10.1186/1471-2229-13-79

**Published:** 2013-05-14

**Authors:** Emma W Gachomo, Jose C Jimenez-Lopez, Sarah R Smith, Anthony B Cooksey, Oteri M Oghoghomeh, Nicholas Johnson, Lamine Baba-Moussa, Simeon O Kotchoni

**Affiliations:** 1 Department of Biology, Rutgers University, 315 Penn St, Camden, NJ 08102, USA; 2 Center for Computational and Integrative Biology, 315 Penn St, Camden, NJ 08102, USA; 3 Department of Biochemistry, Cell and Molecular Biology of Plants, Estación Experimental del Zaidín, Consejo Superior de Investigaciones Científicas (CSIC), Profesor Albareda 1, Granada E-18008, Spain; 4 Department of Biochemistry, University of Abomey-Calavi, Cotonou, Benin

**Keywords:** Angustifolia, Cell morphogenesis, *Arabidopsis thaliana*, Abiotic stress, Biotic stress, T-DNA knockout mutant

## Abstract

**Background:**

ANGUSTIFOLIA (AN), one of the CtBP family proteins, plays a major role in microtubule-dependent cell morphogenesis. Microarray analysis of mammalian AN homologs suggests that AN might function as a transcriptional activator and regulator of a wide range of genes. Genetic characterization of *AN* mutants suggests that AN might be involved in multiple biological processes beyond cell morphology regulation.

**Results:**

Using a reverse genetic approach, we provide in this paper the genetic, biochemical, and physiological evidence for ANGUSTIFOLIA’s role in other new biological functions such as abiotic and biotic stress response in higher plants. The T-DNA knockout *an*-*t1* mutant exhibits not only all the phenotypes of previously described *angustifolia* null mutants, but also copes better than wild type under dehydration and pathogen attack. The stress tolerance is accompanied by a steady-state modulation of cellular H_2_O_2_ content, malondialdehyde (MDA) derived from cellular lipid peroxidation, and over-expression of stress responsive genes. Our results indicate that ANGUSTIFOLIA functions beyond cell morphology control through direct or indirect functional protein interaction networks mediating other biological processes such as drought and pathogen attacks.

**Conclusions:**

Our results indicate that the *ANGUSTIFOLIA* gene participates in several biochemical pathways controlling cell morphogenesis, abiotic, and biotic stress responses in higher plants. Our results suggest that the *in vivo* function of plant ANGUSTIFOLIA has been overlooked and it needs to be further studied beyond microtubule-dependent cell morphogenesis.

## Background

In plant cells, the overall developmental fate, whether it be height, fitness, perception of light, the efficiency of gas exchange or the response to both abiotic and biotic stress conditions, depends ultimately on well programmed cellular morphology, size, expansion, number of cellular constituents and a proper coordination of the cells. The overall rate of cell division, elongation, and cell communication contributes to the final shape of the cells [1,2]. In order to understand plant growth development and improve plant response to a wide range of environmental stresses it is imperative to uncover the genetic interactions and biochemical mechanisms that govern biological processes of cell shape/morphology at different stages of development. Although progress has been made in defining genetic interactions controlling the morphology of specific cell types such as trichomes (leaf hairs) and root hairs [3,4], the cellular mechanisms controlling plant response to a wide range of stress conditions remain elusive.

Recent genetic studies of Arabidopsis *MICROTUBULE* associated mutants including *ANGUSTIFOLIA* (*AN*) and *ZWICHEL* (*ZWI*) revealed widespread cell morphological defects [5-7]. Several mutations of the genes affecting leaf morphology have been reported. However, the mutation affecting the *ANGUSTIFOLIA* gene was shown to result in narrow cotyledons, narrow rosette leaves, twisted seed pods (siliques) [8], and less-branched trichomes [5], suggesting that the *AN* gene might play a role in leaf blade development. The narrow-leaf mutant, *angustifolia* (*an*), was originally isolated from irradiated seeds [8] and this mutation was later used as a visible marker for genetic mapping of *ANGUSTIFOLIA*[9]. To understand the functions of the *AN* gene, *AN* orthologs from various plant species including *Arabidopsis*, Japanese morning glory, rice, moss, and liverwort, have been recently studied [10,11]. Previous sequence comparison studies demonstrated that the plant *AN* gene encodes a protein related to C-terminal binding protein/brefeldin A ADP-ribosylated substrate (CtBP/BARS) with an important role in animal development [12,13], and an encoded protein thought to repress transcription in a manner similar to that of animal CtBPs [6,10]. However, its function has been confirmed to be distinct from that of animal CtBP [14]. All plant AN proteins have LxCxE/D and NLS motifs that are not found in animal CtBPs [6,10]. Moreover, no region corresponding to the long C-terminus of the plant *AN* genes has been detected in animal CtBPs; therefore, the C-terminal region is thought to be related to a plant-specific function of *AN* genes [10]. This suggests that the plant AN proteins may share some evolutionarily conserved functions with invertebrate and vertebrate CtBPs but also possess some unique functions.

So far, there is no other reported biological function of ANGUSTIFOLIA beyond the microtubule cytoskeleton mediated cell morphogenesis in plants. Meanwhile, a transcriptional role of *ANGUSTIFOLIA* similar to that of the invertebrate and vertebrate CtBP family proteins has been suggested on the basis of microarray analysis of transcription in *angustifolia* mutant background [10]. This analysis suggests that the *AN* gene might regulate gene expression as a transcriptional repressor. In addition, the microarray analysis has indicated that several genes were expressed at higher levels in *angustifolia* mutant plants than in wild type [10], suggesting that *ANGUSTIFOLIA* may regulate leaf morphogenesis and other biological processes (i.e., association with microtubule cytoskeleton and by transcriptional regulation). In contrast, recent findings suggest that ANGUSTIFOLIA functions outside the nucleus to control cell morphogenesis [15]. This intriguing finding [15] and previously reported data could co-exist if ANGUSTIFOLIA employs two different molecular mechanisms: one to control cell morphology and the other, biological functions, respectively. We therefore checked this work for other possible biological functions of ANGUSTIFOLIA that have not been reported in plants so far. ANGUSTIFOLIA is an evolutionarily conserved protein representing a perfect model to study functional regulatory network across species.

In this work, we used a reverse genetics approach to examine and characterize a SALK_T-DNA knockout *angustifolia* mutant (*an-t1*) with respect to a wide range of biological phenotypes. Particular attention was paid to newly identified phenotypes. Our data reveal for the first time, new biological functions for *ANGUSTIFOLIA* in plant response to both abiotic and biotic stress conditions. The newly identified phenotypes associated with *ANGUSTIFOLIA* knockout mutation is involved in ROS (H_2_O_2_) and stress responsive gene regulation mediating environmental stress response in higher plants. This phenotypic observation suggests reconsidering the role of ANGUSTIFOLIA beyond cell morphogenesis control.

## Results

### Molecular characterization of an-t1 knockout mutant

**Figure 1:**
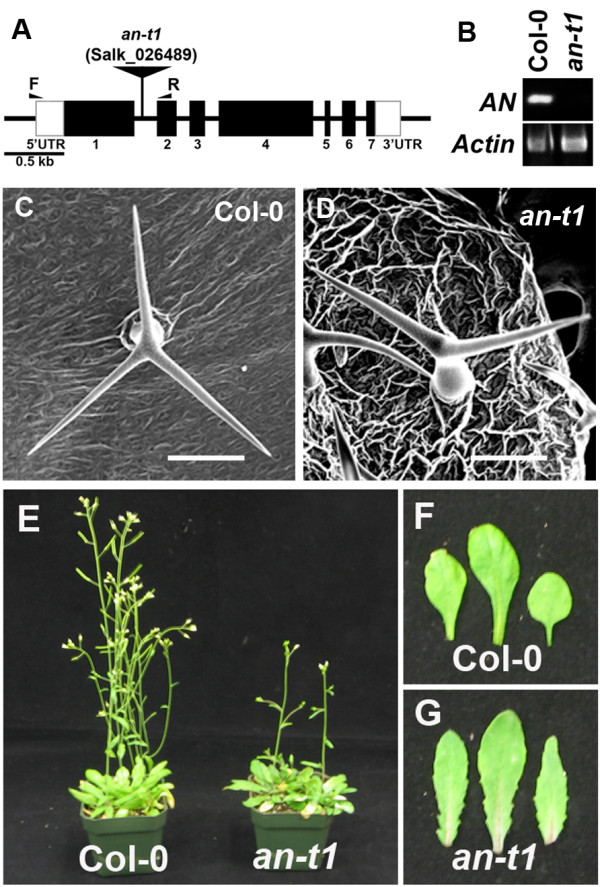
**Physical map of *AN *knockout gene and phenotypic characterization of *an***-***t1 *mutant.** (**A**) The *AN* gene with the positions of exons (numbered black rectangles) and introns (thick lines) are represented. The 5’ and 3’ untranslated regions are depicted in white rectangles. The location of the *an*-*t1* T-DNA insertion is shown using an inverted black triangle. The names and locations of primers used for RT-PCR experiments are also indicated. Bar = 0. 5 kb. (**B**) The T-DNA insertion causes a knockout expression of the gene. The quality of the RNA and the loading control was assayed by monitoring ACTIN gene expression. (**C**-**D**) SEM images of upper developing leaves, showing a mature trichome with three branches in wild type (**C**) and two branches in *an*-*t1* (**D**) plants. (**E**) Wild type and *an*-*t1* plants at seeding stage. *an*-*t1* is dwarf compared to WT col-0 plants. (**F**-**G**) leaf shape structures of wild type (**F**) and an-t1 mutant (**G**) are represented. Bars = 50 μm (**C**, **D**).

ANGUSTIFOLIA (AN) is known to regulate polarized expansions of leaf cells and leaf hair (trichome) branching via the microtubule cytoskeleton [6,10]. In this study, we further investigate the role of ANGUSTIFOLIA in other biological processes. We employed a reverse genetic approach using the *Arabidopsis* T-DNA SALK lines mediating loss of function of *AN* gene to examine new *an*-knockout phenotypes. The SALK_026489 (*an*-*t1*) line harboring a T-DNA insertion in the first intron of the *AN* gene (Figure 1A) was selected and confirmed as null mutant with loss of AN function. We additionally confirmed the location of the T-DNA using the T-DNA-specific oligonucleotide primer LB1 and the *AN*-specific primer Salk_026489-R and examined the *AN* mRNA transcript levels in wild type and *an*-*t1* mutant using RT-PCR. As shown in (Figure 1B), we confirmed that the T-DNA insertion caused a knockout of *AN* gene in *an*-*t1* mutant. Up to date, a T-DNA knockout line of *AN* gene had not been characterized. We next proved that the *an*-*t1* mutant is really an *angustifolia* null mutant, by comparing its phenotype to previously well characterized *angustifolia* null mutants. When compared to the wild type (Figure 1C), the homozygous *an*-*t1* mutant displayed a typical phenotype with a reduced trichome branch number (Figure 1D) -- identical to all previously reported *an*-null mutants [6,10]. The *an*-*t1* is a recessive mutant, and the progeny of the heterozygous plants segregated with the expected 3:1 wild type:mutant Mendelian ratio (110 wild types to 36 *an*-*t1* mutants). We next confirm that the *an*-*t1* reduced-trichome-branch phenotype was indeed caused by the described T-DNA mutation on the *ANGUSTIFOLIA* gene; we performed a complementation test by crossing our *an*-*t1* with a previously characterized *angustifolia* allele (*an*-*1*) [15], yielding 100% (n = 18 seeds) of F1 progeny with reduced trichome branch numbers.

**Figure 2:**
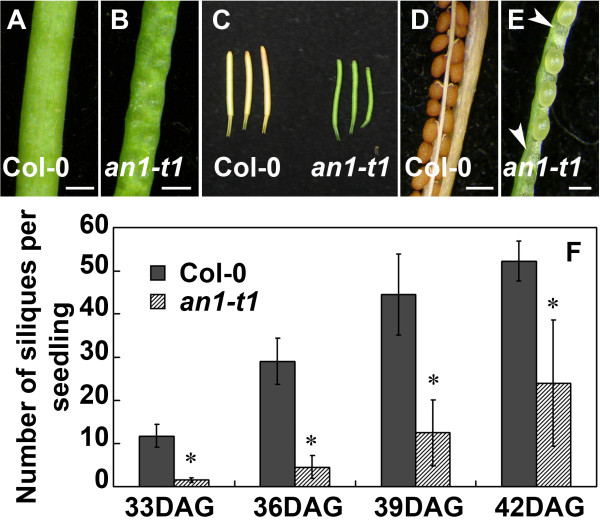
***AN *knockout gene causes irregular seed pods, delay seed maturation and reduced silique number.** (**A-B**) Seed pod pattern of wild type (**A**) and *an*-*t1* (**B**) is represented. (**C**) At the same day after germination, the silique of wild type is maturing faster than that of *an*-*t1*. (**D-E**) At the same day after germination, the seeds of wild type are regularly arranged in the pod and maturing faster (**D**) than the seeds of *an*-*t1* with irregular arrangement (having gaps: see arrows) and still green (**E**). (**F**), The total number of siliques per plant is higher in wild type than in *an*-*t1*. Asterisks indicate significant differences between mutant and wild type. *P <0.05, Student’s t test.

As expected, the mutation also causes a general defect in cell expansion, leading to dwarfism, (dwarf mutants) (Figure 1E), narrower leaf shapes and thickness of leaves (characteristics of all *an*-null mutants) in homozygous *an*-*t1* compared to the wild type as expected (Figure 1F, G). Interestingly, we noticed a significant delay in flowering time of *an*-*t1* mutants demonstrated by a higher number of rosette leaves (15 ± 0.8, n = 10) compared to the wild type (10 ± 0.5, n = 12) at bolting time. The loss of AN function is known to affect various organs leading to twisted seed pods (siliques). As expected, the *an*-*t1* siliques were twisted compared to the wild type (Figure 2A,B). The mutation caused a significant seed maturation delay (Figure 2C-E). The twisted shape of *an*-*t1* siliques was due to an irregularity in the seeds’ formation (presence of gaps in the seed pods) in the *an*-*t1* siliques compared to the wild type (Figure 2D,E). The mutation leads to a reduced number of seed pods per plant when compared to the wild type (Figure 2F).

We next assessed the degree of sequence conservation of AN across monocot and dicot plants including the newly sequenced plant genomes. Our data revealed a high amino acid sequence identity of AN proteins across monocot and dicot plants (Additional file 1: Figure S1), indicating a functional conservation of AN across plant species.

### Novel morphological phenotype of angustifolia null mutant

**Figure 3:**
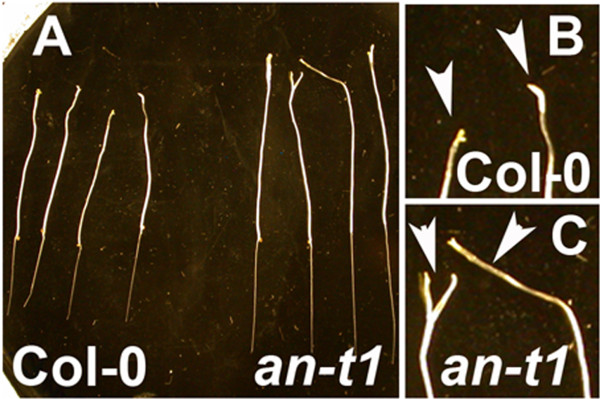
**ANGUSTIFOLIA functions in light and dark-grown seedlings.** (**A-C**) 7 day-after germination (DAG) of dark-grown wild type and *an*-*t1* seedlings (**A**). (**B**) High magnification of dark-grown wild type shoots, (**C**) High magnification of dark-grown *an*-*t1* shoots. Arrow heads indicate the dark-grown hypocotyls and petiole phenotype in WT and *an*-*t1* respectively.

ANGUSTIFOLIA is not essential for cell viability, as *an*-*t1* mutant is perfectly able to grow and produce seeds (Figure 1E). However, our results indicate that ANGUSTIFOLIA appears to be important to other up to now unknown interesting cell morphological phenotypes. We found an additional dark-grown phenotype associated to *an*-*t1* mutant (n = 36 seedlings) (Figure 3). The enhanced cotyledon petiole elongation has only been reported in *wave* and *arp2*/*3* mutants [16,17]. We found that dark grown *an*-*t1* plants display a strong and significantly different hypocotyl and petiole phenotype (Figure 3A,C) compared to wild type (n = 36 *an*-*t1* seedlings and n= 24 WT seedlings) (Table 1). We checked the phenotype in the presence and absence of sucrose to rule out the effect of sugar. Our data indicate that the *an*-*t1* dark-grown phenotype is not a result of sugar effect. In addition, the progeny of heterozygous WT plants segregated with the expected 3:1 wild type:mutant Mendelian ratio under dark-grown conditions, and our complementation test (*an*-*t1* x *an*-*1*) gives a 100% (n = 14 seeds) of F1 dark-grown phenotypic population. The enhanced elongation of petiole in dark-grown *an*-*t1* seedlings suggests that ANGUSTIFOLIA negatively regulates growth in the petiole elongation. To be sure that this new phenotype is not restricted to our *an*-*t1* mutant, we checked and confirmed this new light/dark phenotype in previously reported *angustifolia* null mutants.

**Table 1:** Dark-grown hypocotyl and petiole phenotype of WT and angustifolia mutants

**Genotypes**	**Hypocotyl length (mm)**	**Petiole**
WT (homozygote)	22.5 ± 0.5 (n = 24) a	-
WT (heterozygote)	21.6 ± 0.3 (n = 18) a	-
*an*-*t1*	16.5 ± 0.6 (n = 36) b	+
*an*-1*	18.2 ± 0.3 (n = 32) b	+

The numbers in the parentheses indicate the number of samples analyzed. The sign plus indicates elongate petioles elongation in the dark, while the sign minus indicates the opposite. Mean values with different letters are significantly different from each other, and mean values with the same letter in the group are not significantly different (P <0.05). * *an*-*1* mutant = a gift from the Tsukaya lab.

The cotyledon petiole and root phenotype of the *an*-*t1* mutant under dark grown conditions resembles the phenotype of *wave* mutants, which are associated with actin cytoskeleton defects [17]. We therefore hypothesized that ANGUSTIFOLIA might regulate petiole shape and root elongation through actin cytoskeleton networks. To test our hypothesis, we analyzed the growth of wild type and *an*-*t1* mutants under latrunculin B (LatB), an actin filament depolymerization drug. Both wild type (n = 26 seedlings) and *an*-*t1*mutants (n = 30 seedlings) are significantly affected by Lat B treatment. However, we did not observe any differences in the effect of Lat B between wild type and mutant seedlings (Additional file 2: Figure S2, AB), suggesting that ANGUSTIFOLIA does not regulate root elongation and cell shape through actin cytoskeleton networks. Treatment of wild type (n = 22 seedlings) and *an*-*t1* mutants (n = 25 seedlings) with Taxol, a microtubule stabilizing drug, significantly affected both cytoskeleton and root elongation in *an*-*t1* background compared to wild type (Additional file 3: Figure S3, A-C), suggesting that ANGUSTIFOLIA controls root length and cell morphology through microtubule cytoskeleton.

### ANGUSTIFOLIA controls other new biological processes

**Figure 4:**
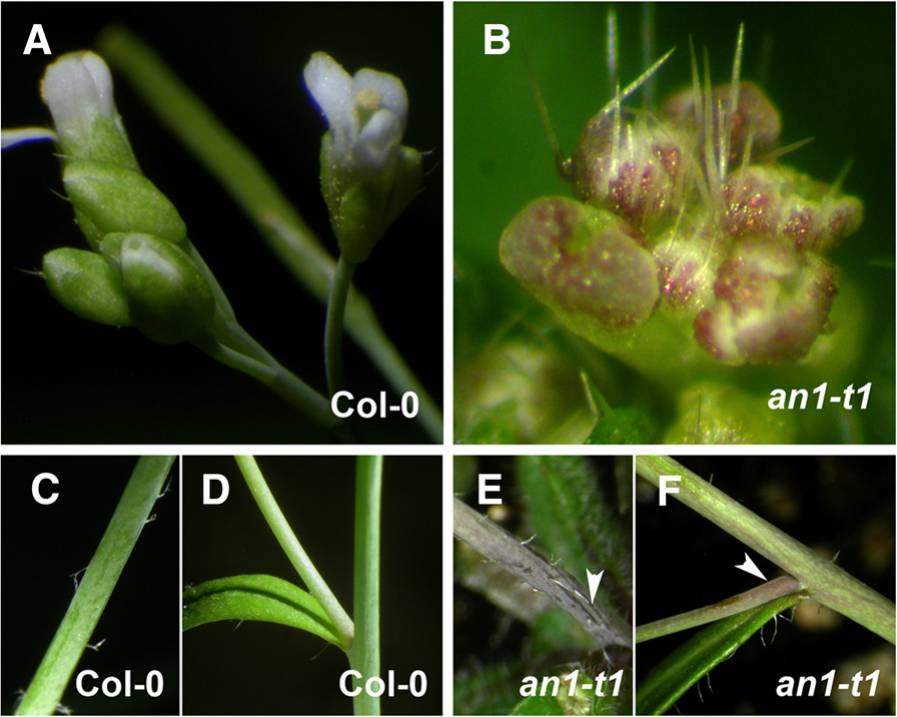
**Mutation in *AN *gene induces accumulation of reactive oxygen species (ROS) in plant specific plant tissues.** (**A-B**) Strong ROS accumulation is depicted in *an*-*t1* flowers (**B**) compared to wild type (**A**) flowers. (**C-F**) Accumulation of ROS in stem (**E**) and branches (**F**) of *an*-*t1* (see arrow heads) compared to wild type stem (**C**) and branches (**D**) that are green.

**Figure 5:**
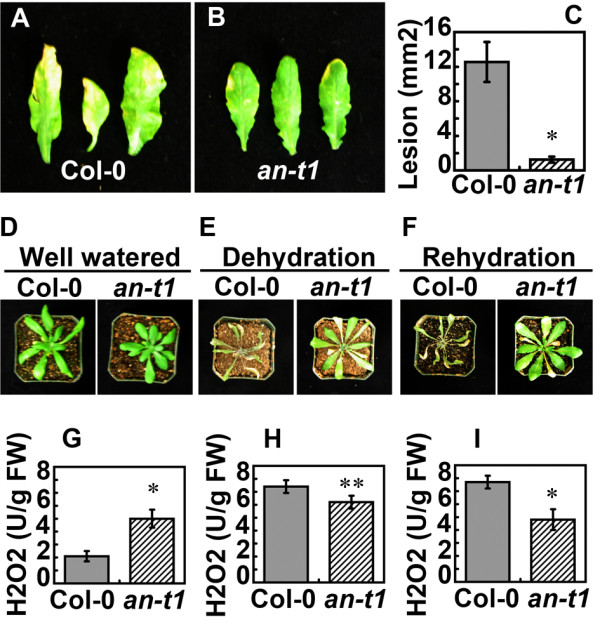
***an-t1 *seedlings cope better with drought stress and pathogen attack than the wild type.** (**A-C**) Phenotypic characterization of wild type and *an*-*t1* under pathogen attack. Pathogenic lesions on wild type (**A**) and *an*-*t1* (**B**) leaves are depicted. (**C**) Quantitative data of pathogenic assay is represented. (**D-I**) Phenotypic characterization of wild type and *an*-*t1* under well watered (**D**), dehydrated (**E**) and re-hydrated (**F**) conditions. The H_2_O_2_ accumulations under well watered (**G**), dehydrated (**H**) and re-hydrated (**I**) are represented. After drought stress (**E**), the *an*-*t1* was able to regain physiological functions upon re-hydration and resume normal growth, unlike wild type that withered away (**F**). H_2_O_2_ content is estimated in U/g FW, where U represents μmol, and FW represents fresh weight. Asterisks indicate significant differences between the mutant and the wild type. *P <0.001, **P <0.05, Student’s t test.

**Figure 6:**
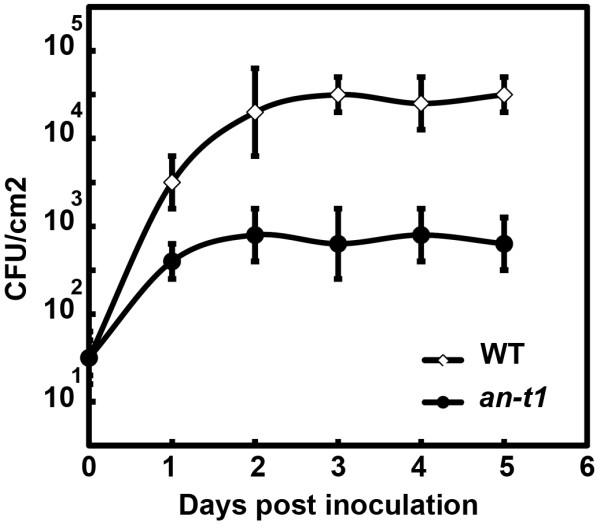
**Responses of the wild type and *an*-*t1 *upon infection with virulent *P. syringae *pv *maculicola *ES4326.** Leaves of 2-week-old plants were inoculated with a bacterial suspension containing 10^5^ CFU (colony forming units) ml^-1^. The growth curve of *P*. *syringae* pv *maculicola* ES4326 in wild type and *an*-*t1* is depicted. The mean values (± SE) of three independent experiments containing four different plants per genotype and per experiment are depicted.

Genetic and biochemical studies have demonstrated the role of ANGUSTIFOLIA in plant cell morphogenesis [6]. However, direct evidence for the role of ANGUSTIFOLIA in other *in vivo* biological functional processes has not been elucidated. We hypothesized that ANGUSTIFOLIA, due to its highly conserved functional status across eukaryotes, might be involved in controlling a wide range of redox regulating and abiotic/biotic related genes. Interestingly, we noticed a higher accumulation of reactive oxygen species (ROS) phenotype in *an*-*t1* mutants compared to the wild type (Figure 4A-D). This ROS accumulation is observed in flower organs (Figure 4B), stems (Figure 4E) and in stem-branching zones (Figure 4F). The overall accumulation of ROS was two times higher in *an*-*t1* compared to wild type (Figure 5G), indicating that ANGUSTIFOLIA negatively regulates the accumulation of cellular ROS. On the other hand, we hypothesized that the high ROS accumulation would predisposed *an*-*t1* to better cope with abiotic and biotic stress conditions compared to the wild type. Indeed, *an*-*t1* mutants (n = 10 seedlings) were more resistant to pathogen attack than wild type (n = 10 seedlings) (Figure 5A-C, Figure 6). To quantitatively estimate the disease resistance phenotype of *an*-*t1*, we assessed the initial rate of bacterial growth, which was found to be similar in wild type and *an*-*t1* (Figure 6). However, at 1 to 3 days post-inoculation, the bacterial titer was approximately 5-fold lower in *an*-*t1* compared to the wild type (Figure 6), suggesting that *an*-*t1* significantly suppresses the pathogen growth better than the wild type. Under drought stress conditions, *an*-*t1* was also able to cope better than the wild type as expected (Figure 5D-I), indicating that ANGUSTIFOLIA regulates various molecular and biological processes beyond the microtubule mediated cell morphogenesis role. Figure 5(G-I) highlights the level of cellular H_2_O_2_ in both WT and *an*-*t1* mutant under normal, drought and re-watering conditions. Under this conditions, only *an*-*t1* was able to regulate the level of endogenous H_2_O_2_ and therefore resume normal growth after re-watering condition compared to WT. In addition, we checked and confirmed that the drought-tolerant and pathogen-resistant phenotypes were also observed in previously reported *angustifolia* null mutants, indicating that these novel phenotypes are not specific to our *an*-*t1* mutant, but rather associated to the *ANGUSTIFOLIA* gene in plants.

### ANGUSTIFOLIA regulates plant stress response through altered stress inducible gene expression

**Figure 7:**
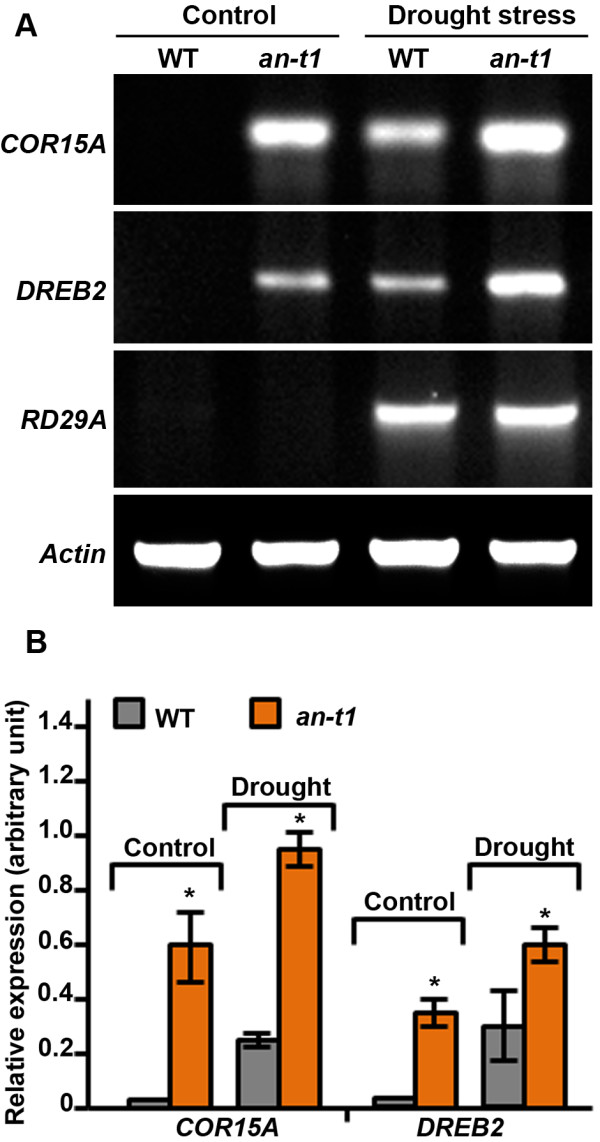
**Stress responsive gene expression analysis in WT and *angustifolia *mutant backgrounds.** (**A**): Plants were grown in soil for one week and drought stress was performed by withholding watering the plant for one week. Leaf material was collected from control and drought stress plants and used in RT-PCR expression analysis as detailed in materials and methods. RD29A (drought inducible gene) was used as positive control for stress condition, while Actin was used as internal control for RT-PCR analysis. (**B**): Quantification of gene expression relative to actin and RD29A transcript in control and drought stress conditions respectively. Expression data (± SE) of three individual samples per genotype are depicted. Asterisks indicate significant differences between the mutant and the wild type (*P <0.05, Student’s t test).

The involvement of the *ANGUSTIFOLIA* gene in abiotic/biotic stress responses is particularly novel. We, therefore, investigated the accumulation of selected abiotic/biotic stress responsive transcripts in WT and *an*-*t1* to elucidate the role of *ANGUSTIFOLIA* in controlling stress response in higher plants (Figure 7A,B). Higher transcript levels of abiotic (drought) stress response genes was observed in the *an*-*t1* mutant compared to WT under both control and drought stress conditions (Figure 7A,B). The osmotic/drought stress response gene, RD29A [18] was used here as the abiotic (drought) stress experimental control, while the actin gene was used as the internal loading control for the RT-PCR experiment.

It is well established that ROS accumulation in plants as a result of stress response often leads to lipid peroxidation [19]. To assess the role of ANGUSTIFOLIA in preventing lipid peroxidation, we analyzed the accumulation of malondialdehyde (MDA)-derived from cellular lipid peroxidation in WT and *an*-*t1* under both control and stress conditions (Table 2). The level of MDA accumulation in *an*-*t1* was significantly lower than that of WT under stress conditions (Table 2).

**Table 2:** Malondialdehyde (MDA) derived from lipid peroxidation in WT and *angustifolia* mutant background under control and stress conditions

**Genotypes**	**MDA (nmol/mg FW)**	
	**Control**	**Drought stress**
WT	21.8 ± 2.5 (n = 16) a	70.5 ± 5.3 (n = 16) b
*an*-*t1*	20.5 ± 1.8 (n = 22) a	32.6 ± 2.5 (n = 22) c
*an*-1*	18.9 ± 2.2 (n = 16) a	35.2 ± 3.8 (n = 16) c

The numbers in the parentheses indicate the number of samples analyzed. Mean values with different letters are significantly different from each other, and mean values with the same letter in the group are not significantly different (P <0.05). * *an*-*1* mutant = a gift from the Tsukaya lab. Drought stress was imposed by withholding watering for one week. FW = fresh weight.

### ANGUSTIFOLIA regulates a wide range of protein networks

ANGUSTIFOLIA has been postulated to function by transcriptional repression [7]. These studies raised the possibility that ANGUSTIFOLIA might also have other unrelated microtubule-associated cytoplasmic functions. Recently, ANGUSTIFOLIA has been reported to function outside the nucleus, and exclusively through membrane trafficking pathways, localizing on punctuate structures around the Golgi [15]. To further understand the function of the *AN* gene beyond microtubule-associated cell morphogenesis regulation, we used the latest high throughput integrated knowledge base Arabidopsis protein interaction network analysis (ANAP) tool [20] to bring insights into the functional interaction protein network of ANGUSTIFOLIA at the cellular level.

ANAP provides a far more detailed, reliable, and extensive knowledge of protein interaction networks than those produced from any single protein interaction database. Additional file 4: Figure S4 (A) shows the resulting network of 10 nodes and 11 edges, based on direct protein interactions. The ANGUSTIFOLIA query protein is marked in red in the center (Additional file 4: Figure S4, A), and each associated protein is linked by a uniquely colored line, based on the interaction detection method, and the rendering rules for the interaction detection methods (Additional file 4: Figure S4, Additional file 5: Table S1).

A more comprehensive interaction analysis using each of the 10 modes (Additional file 4: Figure S4, A) revealed an extended interaction network of 144 nodes and 234 edges (Additional file 4: Figure S4, B), where the initial 10 nodes are marked in red in the centers of the extended interactions (Additional file 4: Figure S4, B). Each associated protein is linked by a uniquely colored line, based on the interaction detection method and the rendering rules from the complete list of all interaction detection methods (Additional file 4: Figure S4, B). This interaction network (Additional file 4: Figure S4, B), which has never been reported before, sheds light into the probable role of ANGUSTIFOLIA beyond microtubule-associated cell morphogenesis control, and argues in favor of ANGUSTIFOLIA functioning in a wide range of biological processes.

## Discussion

ANGUSTIFOLIA is here shown to be important not only for cell shape, trichome, and branching phenotype but also for various other biological functions including environmental stress response in plants. Our data reveals that mutations in the *AN* gene results in delayed flowering, senescence, and reduced seed productivity. Some of these phenotypes are new and the mechanism by which ANGUSTIFOLIA regulates transition processes from the vegetative to the reproductive phase is still unknown. The role of plant ANGUSTIFOLIA at the transcriptional level is still controversial. *ANGUSTIFOLIA* encodes a protein with a sequence similar to CTBPs/BARS that are expressed in all organs [6,15]. CtBP family proteins predominantly function as transcriptional factors. We postulate that ANGUSTIFOLIA might primarily function as a transcription factor modulating the expression of a wide range of genes involved in different biological processes in plants. CTBPs in *Drosophila* have been shown to bind transcriptional repressors such as zinc-finger transcription factors [21]. Although, plant ANGUSTIFOLIA failed to complement *Drosophila* CtBP mutation, [14] uur ANAP database analysis suggested that ANGUSTIFOLIA might still act at the transcription level to control a wide range of biological processes in plants. Microarray analyses showed that transcript levels of ~10 genes in *an*-mutant plants were three time higher than those in the WT plants. As mentioned above, the function of ANGUSTIFOLIA has been associated only with polarized cellular growth and cell morphogenesis through microtubule cytoskeleton accessory proteins [6]. To our knowledge, this is the first time that ANGUSTIFOLIA has been shown to control environmental stress response in higher plants.

The *an*-*t1* dark-grown phenotype (enhanced petiole elongation and a premature activation of leaf initiation (Figure 3A-C, Table 1)) is a very new phenotype. In dark-grown WT, petiole elongation and leaf initiation are normally inhibited (Figure 3A-C, Table 1). Other previously distorted mutants that displayed this dark-grown phenotype include *brk1*, *scar1*,*2*,*3*,*4* quadruple, and *dis2*/*arpc2* mutants [17]. The dark-grown phenotype has been also reported in the *LEAFY COTYLEDON1* mutant [22]. Mutation of the *LEAFY COTYLEDON1*, a putative transcription co-activator, causes similar petiole and hypocotyl dark-grown phenotypes [22] suggesting that this phenotype is under a developmental-dependent regulation pathway [17,22]. Furthermore, the newly identified dark-grown phenotype indicates that ANGUSTIFOLIA negatively regulates growth in the petiole [16,17].

In addition, the results presented here demonstrate that plants with a loss of function of ANGUSTIFOLIA were more tolerant to drought and pathogen attack than WT plants (Figure 5A-F). The fact that this knockout mutant line is more tolerant than the WT suggests that the expression of the *AN* gene co-represses the abiotic and biotic associated genes. In general, no apparent phenotypic difference was observed in *an*-*t1* and WT plants under non-stress conditions, indicating that the activity of ANGUSTIFOLIA is stress inducible.

In order to further ascertain the role of the *AN* gene in plant stress tolerance, the expression profile of stress-related genes was analyzed in both WT and *an*-*t1* under control and stress conditions (Figure 7A,B). The results from the expression analysis of DREB2 (member of drought responsive element binding subfamily A-2 transcription factor family) and COR15 (cold-regulated 15-A) genes show higher transcript accumulation on *an*-*t1* background under both control and stress conditions (Figure 7A,B). This demonstrates that the *AN* gene negatively regulates stress-inducible genes and controls the stress response in higher plants. These findings are consistent with the suggestion that the *AN* gene controls the stress response in plants at the transcriptional level by regulating the expression of a selected-stress-responsive and ROS-relates genes as suggested by the microarray and the interaction protein network data. The role of the *AN* gene in stress response is further corroborated by the accumulation of higher MDA derived from cellular lipid peroxidation in WT compared to *an*-*t1* and *an*-*1* mutants under stress conditions (Table 2).

Similar to the dehydrogenase protein superfamily, CtBP family proteins, the mammalian ANGUSTIFOLIA homologs, contain a conserved Rossman fold motif required for the binding of nicotinamide adenine dinucleotide co-factors [23]. Indeed, CtBP1 and CtBP2 have been shown to bind to NAD^+^/NADH redox status [24,25], enhancing the interaction of CtBP with target transcription factors [25]. This redox status on modulating the repression versus activation of transcription by CtBP is an interesting aspect for future investigation. Since CtBP appears to be a redox-sensing transcriptional regulator, its activity may be modulated by the energy status of the cell during development and in diseases or abiotic stress conditions. The *An*-*t1* knockout line displayed an elevated level of ROS (H_2_O_2_) under drought and pathogen attack (Figure 5G-I). This is an interesting observation because the generation of ROS and related molecules is common to both abiotic and biotic stress [26]. Both types of stress trigger the increase of H_2_O_2,_ probably via common mechanisms. The probability of ANGUSTIFOLIA playing a role in pathogen defense and abiotic stress response has not been reported before. The stress-activated ANGUSTIFOLIA could possibly have a second role. Besides the ROS-mediated redox activity, it may catalyze reactions that can be considered as metabolic escape routes providing alternative pathways for NAD(P)H under various environmental stress conditions. Indeed, ANAP database analysis showed that ANGUSTIFOLIA interacts with several oxidation-reduction biological processes and abiotic and biotic stress response processes (Additional file 4: Figure S4, B, Additional file 6: Table S2) supporting the stress response phenotype observed in this study.

## Conclusions

In conclusion, the present study provides a substantial body of work that demonstrates the potential of ANGUSTIFOLIA to regulate cell morphogenesis and to confer both abiotic and biotic stress tolerance through ROS-mediated redox activity. It would be interesting to investigate the *ANGUSTIFOLIA* knockout genes in agronomically important crops with the aim of improving crop tolerance to multiple environmental stressors. Further studies at transcriptional and proteomic levels are needed to elucidate the wide range of pleiotropic biological functions of ANGUSTIFOLIA in plants.

## Materials and methods

### Plant material, growth conditions, and stress treatments conditions

*Arabidopsis thaliana* (ecotype Col-0) and *an*-*t1* knockout mutant (T-DNA SALK_026489) from Arabidopsis Biological Research Center (ABRC) were used throughout this work. Appropriate seeds were sown on Murashige and Skoog (MS) agar plates or soil and seedlings were allowed to grow under continuous illumination (120–150 μEm^−2^s^−1^) at 22°C. Dark-growing conditions were obtained by wrapping the plates with three layers of aluminum foil and the plates were incubated under the same growth conditions. For stress conditions, seedlings were used directly from MS-agar plate and soil-grown seedlings were subjected to cytoskeleton associated drugs (LatB and Taxol) and abiotic/biotic stress treatments respectively. For dehydration stress, 2-week-old soil-grown plants were kept without watering for pre-determine time period and then re-watered according to Kotchoni *et al*. [19]. For pathogen infection, 2-week-old soil seedlings were infected with *Pseudomonas syringae* as described by Barth *et al*. [27] (See detail, below).

### Infection of plants with virulent pseudomonas syringae pv maculicola ES4326

For pathogen infection, 2-week-old soil seedlings were infected with *Pseudomonas syringae* pv *maculicola* ES4326 as described by Barth *et al*. [27]. At the indicated time, bacterial growth in leaves was determined in 0.55 cm^2^ leaf discs that were extracted by macerating the discs in 300 ml of 10 mM MgCl_2_. Serial dilutions were plated out on agar plates containing 100 mg ml^-1^ streptomycin. The infection experiment was carried out in three independently replicate experiments.

### Mutant characterization and reverse transcription (RT)-PCR analysis

T-DNA insertion in the *ANGUSTIFOLIA* gene was PCR-confirmed using *ANGUSTIFOLIA* gene specific primers (For: 5’-TACAACAACCCAAGTGGAAGA-3’; Rev: 5’-TCGAGGGCCTGATTCGTTCTT-3’) and T-DNA left border primer Lb: 5’-CCGTCTCACTGGTGAAAAGAA-3’. The expression of the *ANGUSTIFOLIA* gene in *an*-*t1* mutant background was analyzed by extracting total RNA from the *an*-*t1* homozygous line using TRIzol reagent (Molecular Research Center) and reverse transcribed as described previously [28]. Thereafter, the cDNA was used as template for PCR using *ANGUSTIFOLIA* gene specific primers. *Actin2*[29] transcripts (*Act2*-For: 5′-GCGGATCCATGGCTGAGGCTGATGATATTCAACC-3′; *Act2*-Rev: 5′-CGTCTAGACCATGGAACATTTTCTGTGAACGATTCC-3′) was used as internal control.

For abiotic/biotic-gene expression analysis, gene-specific primers (Table 3) were used in RT-PCR analysis to run 20 or 25 amplification PCR cycles (linear range of amplification) unless otherwise noted. The linear range of amplification was determined by running increasing cycle numbers and analyzing the amount of cDNA fragments [28]. PCR fragments were separated on 1% agarose gels containing ethidium bromide. ACTIN gene was used as internal control [29]. For quantitative expression analysis, RT-PCR was performed in triplicate for each gene as described above and band intensities of the three replicates were respectively quantified with ImageQuant 5.0 (Amersham Biosciences). Fragments generated from ACTIN served as internal controls, while fragments of *RD29A* gene served as drought stress control in both wild type and *an*-*t1* mutant. Specifically, the mean values of pixel intensity of the bands (*COR15A* and *DREB2* genes) were normalized against (i) ACTIN band intensity under non-stress (control) condition, and against (ii) *RD29A* pixel band intensity under drought stress condition.

**Table 3:** Sequences of oligonucleotide primers used for stress-responsive gene expression analysis

**Sequence description**	**Sequence**	**Number of bases**	**AGI**
DREB2-F	TCGAGGAAAGGTTGTATGAGAGG	23	AT1G75490
DREB2-R	AGAGTCGCTGCTGCTTTTTC	20	AT1G75490
COR15A-F	ACTCAGTTCGTCGTCGTTTCTCAA	24	AT2G42540
COR15A-R	GTTTGCGGCTTCTTTTCCTTTCT	23	AT2G42540
RD29A-F	ATGAGAATGGTGCGACTAA	19	AT5G52310
RD29A-R	CGTTGACCTTCCGTTGACCA	20	AT5G52310

### ANAP database dependent ANGUSTIFOLIA protein interaction network

To fully understand the role of *ANGUSTIFOLIA* at the molecular and cellular level in various biological processes we used the newly developed protein interaction network database ANAP (http://gmdd.shgmo.org/Computational-Biology/ANAP/ANAP_V1.1) [20]. To construct the protein interaction network, the ANAP database tool utilized data from multiple sources, comprising both predicted interactions and experimentally tested evidence [20].

### Determination of flowering time

Flowering time was assessed by counting the number of rosette leaves when flower bolts were 1 cm in length or when floral buds were visible at the center of the rosette as previously reported [28].

### Measurement of H_2_O_2_ content

Rosette leaves (approximately 0.1 g) were incubated under rocking condition (250 rpm) in 3 ml reagent [25 mM phosphate buffer pH 7.0; containing 0.05% guaiacol (Sigma) and 2.5 units ml^-1^ horseradish peroxidase (Sigma)] in darkness at 25°C for 2 h. Absorbance of the solution was measured at 450 nm as described [30]. H_2_O_2_ concentrations were determined using a H_2_O_2_ standard curve, containing 5, 10, 25, 50, 75, 100, or 150 mM H_2_O_2_ (Sigma) as previously described [28].

### Lipid peroxidation assay

The levels of lipid peroxidation in plant cells were assayed with the thiobarbituric acid (TBA) test, which determines the amounts of malondialdehyde (MDA) as end product of lipid peroxidation [19].

### Statistical analysis

Experiments were performed at least three times. Data were expressed as mean values ± SE. P values were determined by Student’s t test analysis.

## Competing interests

The authors declare that they have no competing interests.

## Authors’ contributions

SOK conceived the study. SOK, EWG JCJ-L and LB-M wrote the manuscript. EWG, JCJ-L, ABC, OMO, NJ and SOK performed the work and analyzed the data. SRS prepared the samples and took the SEM images and analyzed the microscopic images. All the authors approved the final manuscript.

## Supplementary Material

Additional file 1: Figure S1 ANGUSTIFOLIA is evolutionary conserved across plant species. Comparison of ANGUSTIFOLIA full length amino acid sequences from different plant species are represented. The asterisks indicate the conserved amino acids across species. phosphorylation motifs detected in AN from Arabidopsis are indicated. Amino acids identical in all ANGUSTIFOLIA sequences are shaded in gray. At: *Arabidopsis thaliana*, Os: *Oryza sativa*, Sb: *Sorghum bicolor*, Zm: *Zea mays*, Mt: *Medicago truncatula*, Gm: *Glycine max*, AN: Angustifolia. Click here for file

Additional file 2: Figure S2 The effect of latrunculin B (LatB) on wild type and *an*-*t1* seedlings is indistinguishable. (A) Light-grown seedling phenotype of wild type and *an*-*t1* at different concentrations of LatB. (B) Root phenotype of wild type and *an*-*t1* at different concentrations of LatB. Bars = 5 mm. Click here for file

Additional file 3: Figure S3 *an*-*t1* knockout mutant is more sensitive to taxol treatment than wild type. (A-B) Light-grown phenotypes of wild type (A) and *an*-*t1* (B) at different concentrations of taxol. (C) Root phenotype of wild type and *an*-*t1* at different concentrations of taxol. Bars = 5 mm. Asterisks indicate significant differences between the mutant and the wild type. *P <0.05, Student’s t test. Click here for file

Additional file 4: Figure S4 ANAP Protein interaction network generated using the ANGUSTIFOLIA protein (AT1G01510). (A) The ANAP framework of the interaction based upon the node relationship of the source database and direct interaction detection method is depicted. (B) A more comprehensive interaction of ANGUSTIFOLIA with several other proteins using a depth search mode (for indirect interaction searches) is generated. Click here for file

Additional file 5: Table S1 The depth search protein interaction network generated using the ANGUSTIFOLIA protein (AT1G01510) as query. The total number of interactions detected by ANAP from experimental data and inference-based approaches supporting the interactions are depicted. The databases supporting the interactions are shown. Click here for file

Additional file 6: Table S2 Selected members of Gene ontology (GO)-enriched protein interaction with Angustifolia clustered by cellular component. These proteins were selected from the extended interaction network (144 proteins) depicted in Additional file 4: Figure S4, B. Click here for file
