# Supplementary figures and images for: The cell morphogenesis ANGUSTIFOLIA (AN) gene, a plant homolog of CtBP/BARS, is involved in abiotic and biotic stress response in higher plants
Source: BMC Plant Biol. 2013 May 14;13:79. doi: 10.1186/1471-2229-13-79 (PMC3663690; doi:10.1186/1471-2229-13-79)

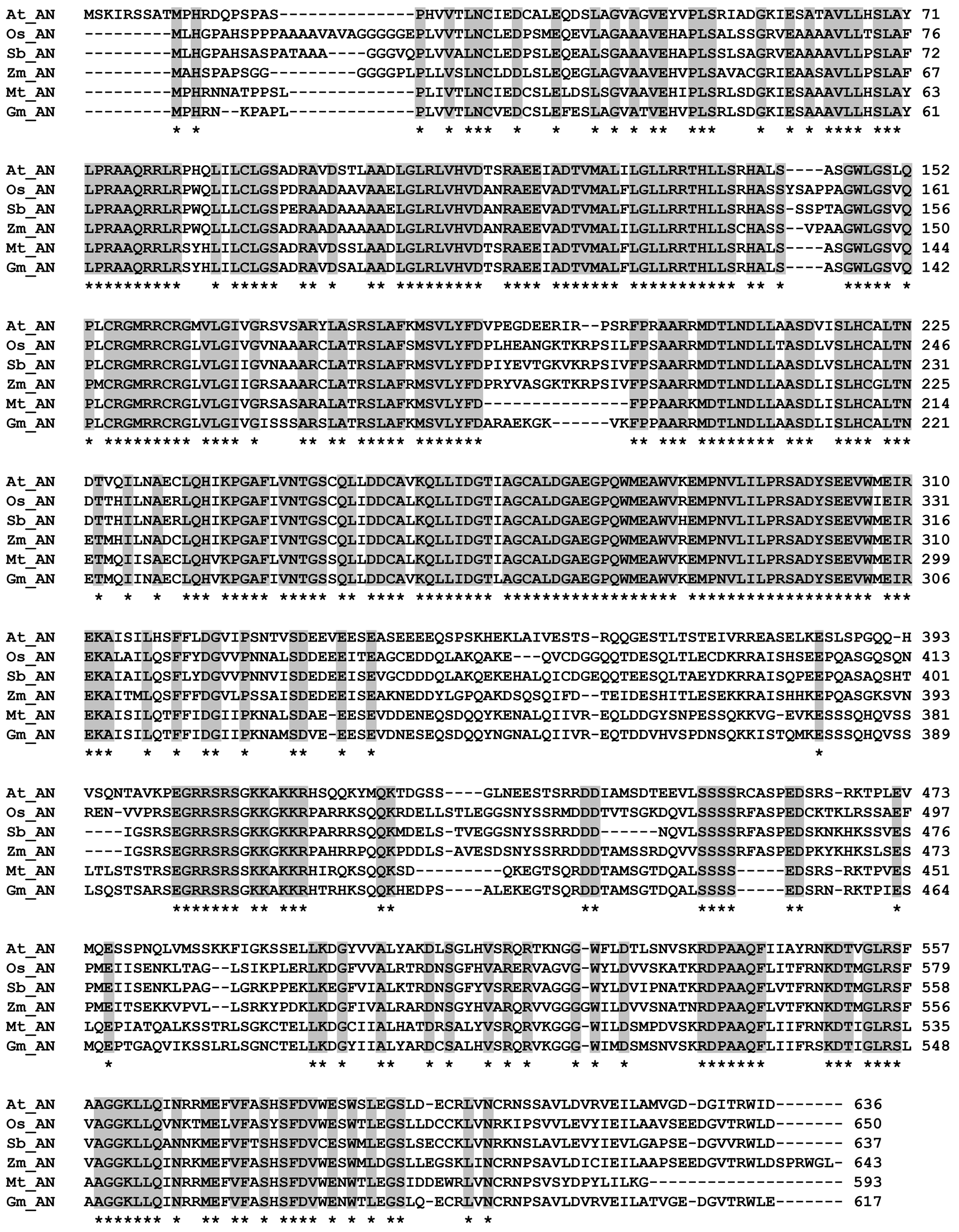

Supplement: Additional file 1: Figure S1 — ANGUSTIFOLIA is evolutionary conserved across plant species. Comparison of ANGUSTIFOLIA full length amino acid sequences from different plant species are represented. The asterisks indicate the conserved amino acids across species. phosphorylation motifs detected in AN from Arabidopsis are indicated. Amino acids identical in all ANGUSTIFOLIA sequences are shaded in gray. At: Arabidopsis thaliana, Os: Oryza sativa, Sb: Sorghum bicolor, Zm: Zea mays, Mt: Medicago truncatula, Gm: Glycine max, AN: Angustifolia. [file 1471-2229-13-79-S1.tiff]

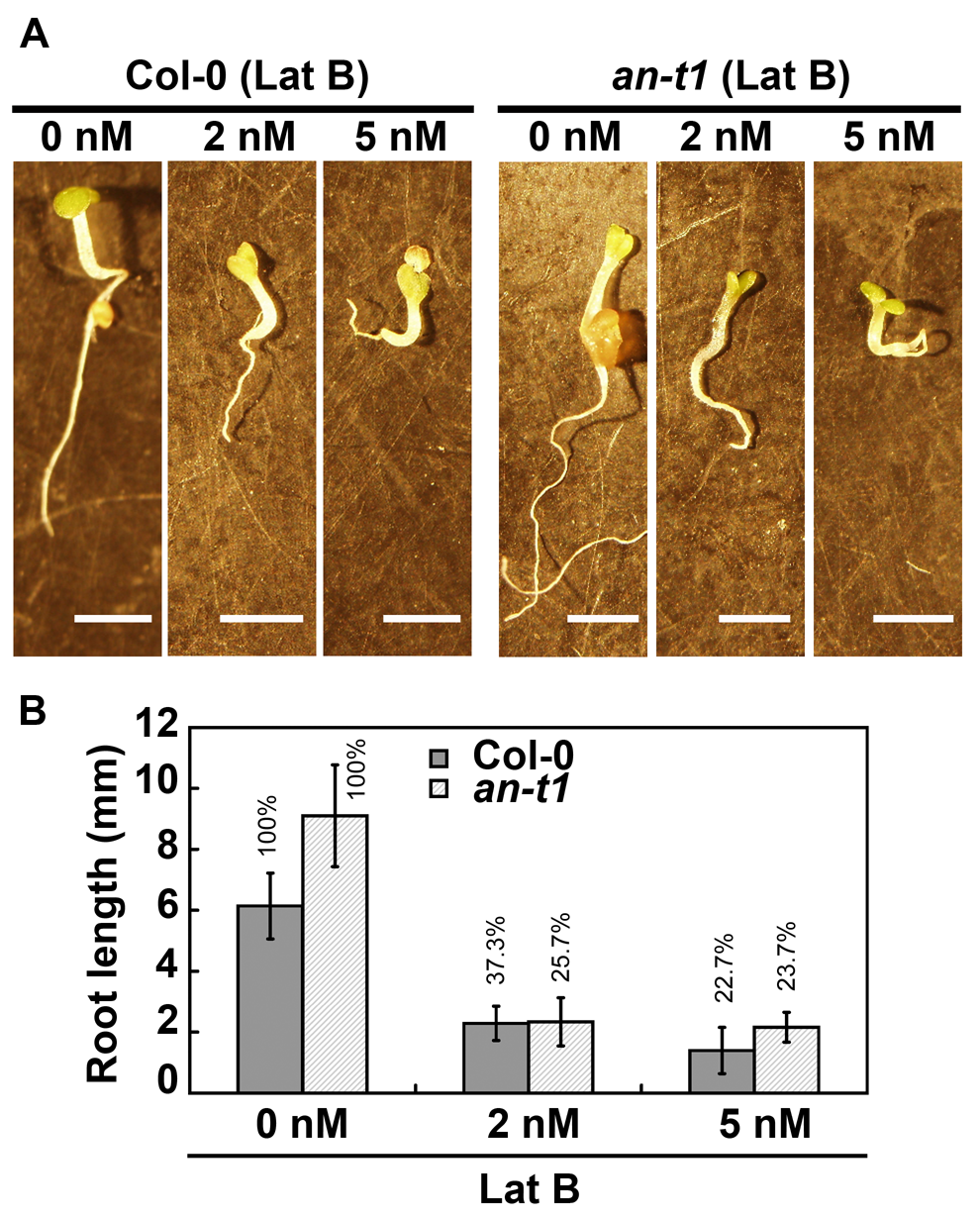

Supplement: Additional file 2: Figure S2 — The effect of latrunculin B (LatB) on wild type and an-t1 seedlings is indistinguishable. (A) Light-grown seedling phenotype of wild type and an-t1 at different concentrations of LatB. (B) Root phenotype of wild type and an-t1 at different concentrations of LatB. Bars = 5 mm. [file 1471-2229-13-79-S2.tiff]

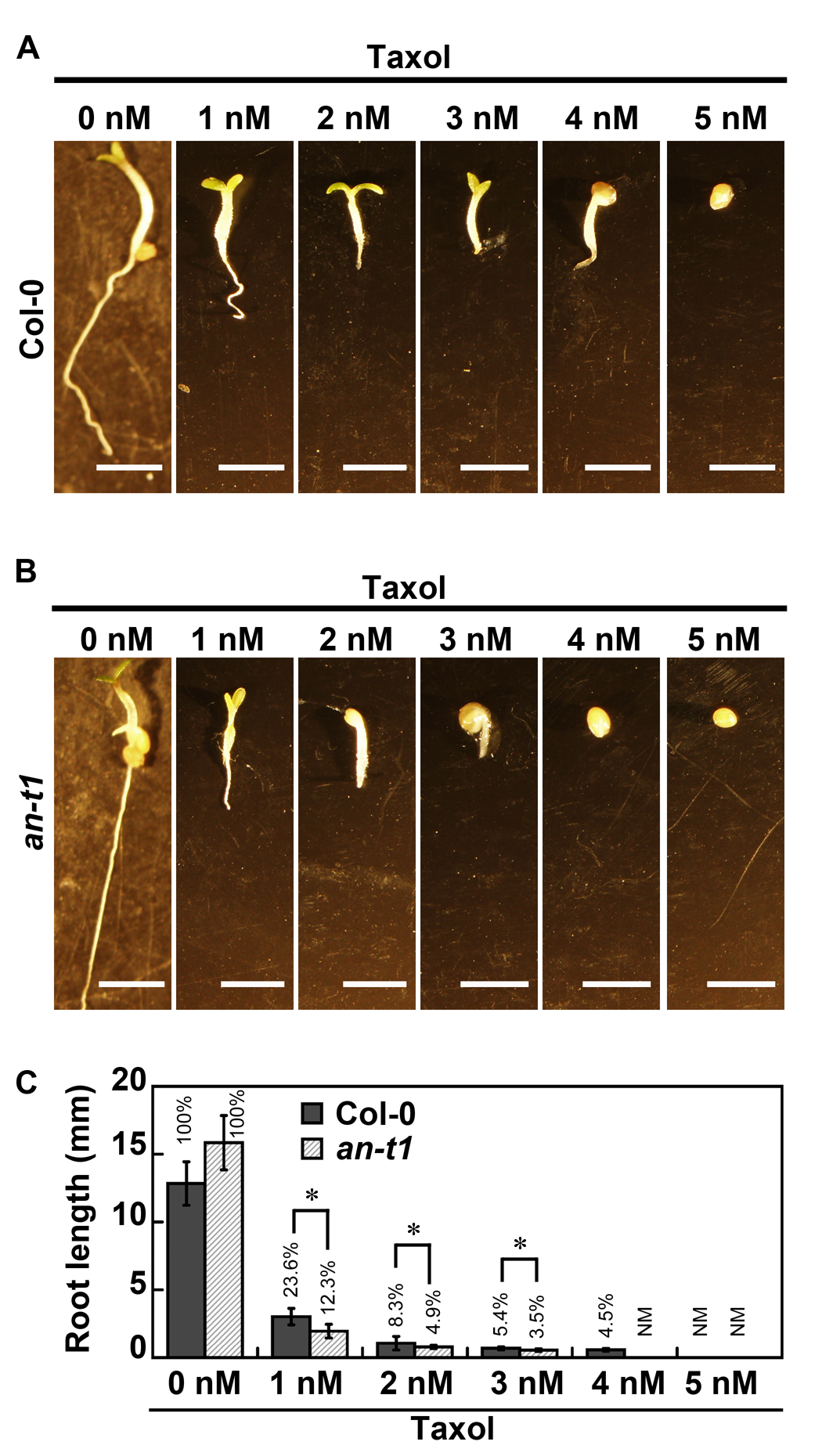

Supplement: Additional file 3: Figure S3 — an-t1 knockout mutant is more sensitive to taxol treatment than wild type. (A-B) Light-grown phenotypes of wild type (A) and an-t1 (B) at different concentrations of taxol. (C) Root phenotype of wild type and an-t1 at different concentrations of taxol. Bars = 5 mm. Asterisks indicate significant differences between the mutant and the wild type. *P <0.05, Student’s t test. [file 1471-2229-13-79-S3.tiff]

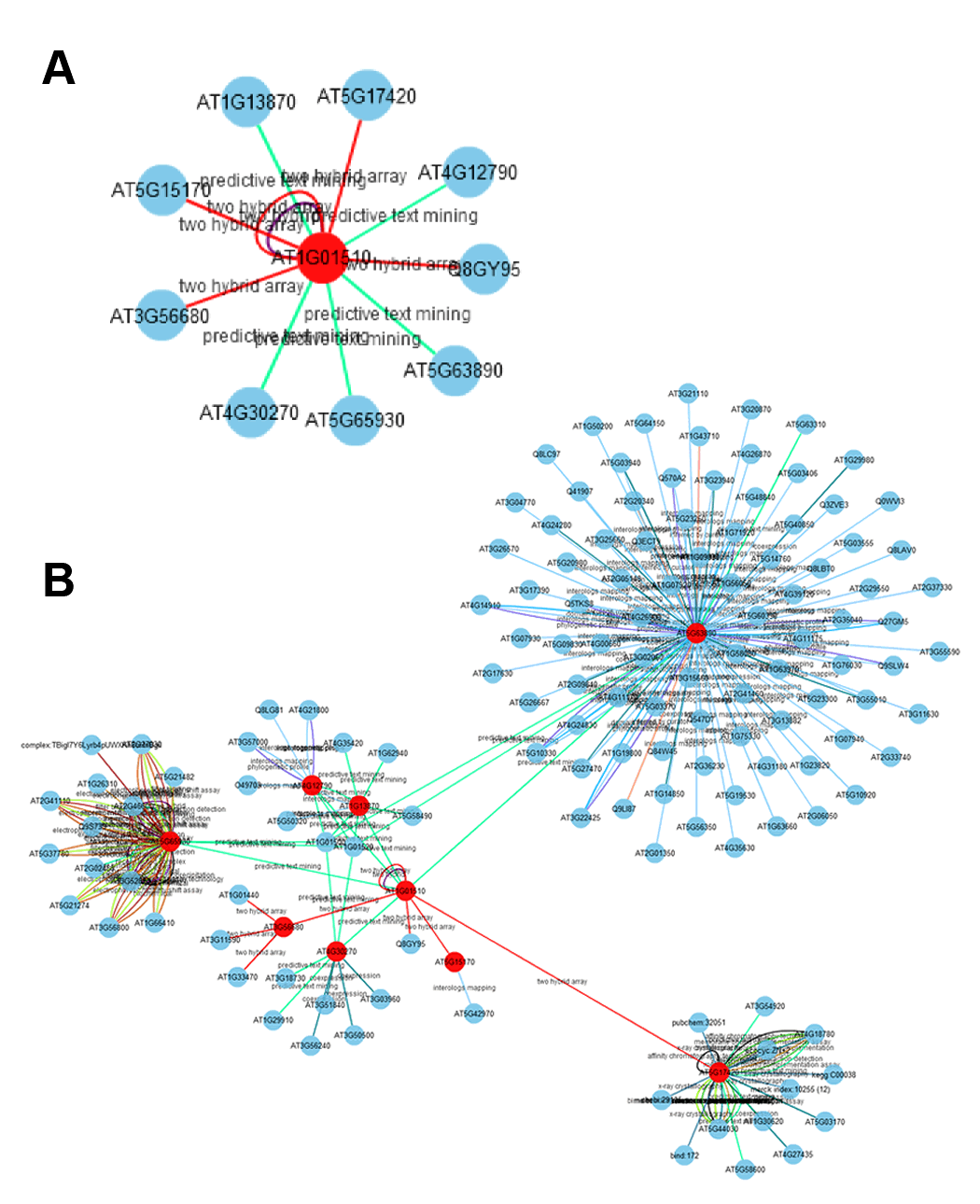

Supplement: Additional file 4: Figure S4 — ANAP Protein interaction network generated using the ANGUSTIFOLIA protein (AT1G01510). (A) The ANAP framework of the interaction based upon the node relationship of the source database and direct interaction detection method is depicted. (B) A more comprehensive interaction of ANGUSTIFOLIA with several other proteins using a depth search mode (for indirect interaction searches) is generated. [file 1471-2229-13-79-S4.tiff]
