# Supplementary material for: The cell morphogenesis ANGUSTIFOLIA (AN) gene, a plant homolog of CtBP/BARS, is involved in abiotic and biotic stress response in higher plants
Source: BMC Plant Biol. 2013 May 14;13:79. doi: 10.1186/1471-2229-13-79 (PMC3663690; doi:10.1186/1471-2229-13-79)
Supplement: Additional file 6: Table S2 — Selected members of Gene ontology (GO)-enriched protein interaction with Angustifolia clustered by cellular component. These proteins were selected from the extended interaction network (144 proteins) depicted in Additional file 4: Figure S4, B. [file 1471-2229-13-79-S6.doc]

**Additional file 2: Table S2:** Selected members of Gene ontology (GO)-enriched protein interaction with Angustifolia clustered by cellular component.

These proteins were selected from the extended interaction network (144 proteins) depicted in Figure S4, B.

| Clustered by GO cellular component | Gene locus | GO Molecular function | GO biological process |
| --- | --- | --- | --- |
| (I)  Nuclear | At1g01520 | ALTERED SEED GERMINATION 4, ASG4 | Regulation of transcription, DNA-dependent, response to salt stress, sequence-specific DNA binding transcription factor activity |
|  | At1g26310 | AGL10, CAL, CAL1, DNA binding | Floral meristem determinacy, positive regulation of flower development, regulation of transcription, DNA-dependent |
|  | At5g37780 | ACAM-1, TCH1 | Gene expression regulation upon a variety of abiotic stimuli, including water spray, subirrigation, wind, touch, wounding, or darkness, response to mechanical stimulus |
|  | At5g15170 | 3'-tyrosyl-DNA phosphodiesterase activity | DNA repair, metabolic process |
| (II)  Vacuolar or Plasma Membrane | At2g27030 | ACAM-2, CAM5 | Calcium ion binding protein, high affinity to kinesin-like calmodulin binding motor protein |
|  | At5g27030 | ZWI, ZWICHEL | ATP catabolic process, metabolic process, pollen germination, trichome branching, Kinesin-like calmodulin binding, microtubule binding, microtubule motor activity, protein binding |
| (III)  Cytoplasm | At4g21800 | QQT2, QUATRE-QUART2 | Cell division, embryo development, microtubule associated protein, ATP binding, nucleotide binding |
| (IV)  Cytosol | At5g58490 | Q9FGH3 | Cellular metabolic process, lignin biosynthetic process, metabolic process, oxidation-reduction process |
| (V)  Cell wall | At4g30270 | Meristem-5, MERI-5, Senescence 4, SEN4, Xyloglucan Endotransglucosylase/Hydrolase 24, XTH24, | Aging, gibberellic acid mediated signaling pathway, metabolic process, plant-type cell wall loosening, response to brassinosteroid stimulus, response to gibberellin stimulus |
|  | At5g17420 | Cellulose Synthase Catalytic Subunit 7, CESA7, Irregular Xylem 3, IRX3, Murus 10, RUR10 | Cellulose biosynthetic process, cell wall thickening, metabolic process, plant-type cell wall biogenesis, rhamnogalacturonan I side chain metabolic process, secondary cell wall biogenesis |
| (VI)  Chloroplast | At5g17420 | Cellulose Synthase Catalytic Subunit 7, CESA7, Irregular Xylem 3, IRX3, Murus 10, RUR10 | Cellulose biosynthetic process, cell wall thickening, metabolic process, plant-type cell wall biogenesis, rhamnogalacturonan I side chain metabolic process, secondary cell wall biogenesis |
